# Utilizing T1-weighted MRI intensity indices to evaluate in-vivo neurotoxicity in a South African cohort with environmental manganese exposure

**DOI:** 10.1016/j.jtemb.2025.127732

**Published:** 2025-08-22

**Authors:** Susan R. Criswell, Irene Faust, Susan Searles Nielsen, Wendy W. Dlamini, Gill Nelson, Jay R. Turner, Joshua S. Shimony, Jason Lenox-Krug, Jordan A. Killion, T. Noah Hutson, Brad A. Racette

**Affiliations:** a Department of Neurology, Barrow Neurological Institute, Phoenix, AZ, USA; b Department of Neurology, Washington University School of Medicine, St. Louis, MO, USA; c Department of Epidemiology, School of Public Health, University of Washington, Seattle, WA, USA; d School of Public Health, Faculty of Health Sciences, University of the Witwatersrand, Parktown, South Africa; e Department of Energy, Environmental, and Chemical Engineering, Washington University, St. Louis, MO, USA; f Mallinckrodt Institute of Radiology, Washington University School of Medicine, St. Louis, MO, USA

**Keywords:** Manganese, MRI, Biomarker, Neurotoxicology

## Abstract

**Background:**

Excessive exposure to manganese (Mn) causes parkinsonism. Occupational Mn exposure is associated with increased T1-weighted globus pallidus signal on magnetic resonance imaging (MRI) secondary to in-vivo Mn deposition.

**Methods:**

The present study evaluated the T1-weighted pallidal index (PI) as an in-vivo marker of Mn exposure and neurotoxicity in chronic environmental Mn exposure. A total of 53 Black South African participants with a range of residential environmental Mn exposures due to proximity to one of the world’s largest smelters underwent T1-weighted MRI.

**Results:**

The PI was associated with parkinsonism as measured by the total Unified Parkinson’s Disease Rating Scale motor subsection part 3 (UPDRS3) in all participants (β=0.10, confidence interval 0.01, 0.18). Further, the PI was positively associated with total UPDRS3 scores (β=0.14, confidence interval 0.03, 0.25) and the lower limb rigidity subscore (β=0.04, confidence interval 0.005, 0.07) among those who regularly consumed alcohol (≥3 drinks/week), but not in those who occasionally consumed alcohol (<3 drinks/week).

**Conclusion:**

Our findings suggest T1-weighted PI is associated with clinical neurotoxicity in environmental Mn exposure. This association is amplified by regular consumption of alcohol.

## 1. Introduction

Globally, millions of individuals are exposed to airborne environmental manganese (Mn) due to the combination of industrial processes like the combustion of fossil fuels, the erosion of soils next to Mn mining operations, and stack emissions from industrial processes like steel-making and smelting [1–3]. Mn is also an essential metal in electric vehicle batteries and is listed as a critical mineral on the Federal Registry [4]. There is a well-established link between occupational Mn exposure and parkinsonism [5–7]. More recently, we have described a similar phenotype in residents of a South African community located immediately adjacent to a large Mn smelter [3,8–10].

The paramagnetic properties of Mn allow for identification of brain deposition with non-invasive magnetic resonance imaging (MRI). Mn^2+^ has five unpaired electrons in the 3rd orbital resulting in a large magnetic moment, causing a shortening of the spin-lattice relaxation time and ultimately an increase in T1-weighted MRI signal intensity [7,11]. Bilateral symmetrical increases in T1-weighted signal in the globus pallidus is the characteristic imaging pattern associated with Mn over-exposure. These changes are commonly assessed with a pallidal index (PI) to quantify Mn accumulation in globus pallidus, defined as the ratio of the signal intensity in the globus pallidus to that in the subcortical frontal white matter multiplied by 100 [12]. The PI is widely used as a semi-quantitative indicator of brain Mn status in human studies [11,13–20] and is associated with Mn exposure [7,18], Mn blood levels [20], and more recently clinical parkinsonism [19]. While the association between occupational Mn exposure and T1 pallidal hyperintensities is well documented, the use of MRI in environmental Mn exposures is limited to a few pediatric studies. Two case studies document increased T1 pallidal signal in children with suspected Mn toxicity secondary to consuming drinking water with high Mn concentrations [21,22]. In comparison, Dion et al. found no difference in the pallidal T1 signal when comparing a cohort of Canadian children exposed to drinking water with high Mn concentrations to children who consumed drinking water with low Mn concentrations [23].

In this study, we examined the utility of T1-weighted MRI in an adult cohort environmentally exposed to Mn. We hypothesized that participants with higher Mn exposure would have a higher T1-weighted pallidal index (PI) and this would be associated with greater severity of parkinsonism as measured by the Unified Parkinson’s Disease Rating Scale motor subsection 3 (UPDRS3) [24] including lower limb rigidity, axial signs, and upper limb bradykinesia as we have observed in occupationally exposed cohorts [5]. Demonstrating an association between an MRI biomarker of Mn exposure and clinically relevant outcomes has important environmental and health implications given that environmental Mn exposures are highest in urban poor communities [25,26]. We tested this hypothesis in a subset of Black participants recruited from a well characterized environmentally Mn-exposed cohort in South Africa [3,8–10].

## 2. Materials and methods

### 2.1. Protocol approvals and participant consents

The University of the Witwatersrand Human Research Ethics Committee and the Washington University Human Research Protection Office approved this study. All participants provided written, informed consent prior to participation.

### 2.2. Participants

We recruited a subset of 53 participants for imaging between 2016 and 2020 from a larger population-based research study (N = 621) focused on a community in Gauteng province, South Africa with long-term environmental Mn exposure [10]. Participants in this community, Meyerton, lived in one of three settlements (Old Sicelo, New Sicelo, or Noldick) located within 5 km of one of the world’s largest Mn smelters. In the larger cohort study, trained field workers recruited research participants by visiting a preselected, population-based sample of homes in each settlement [10]. Participants completed clinical assessments and provided toenail samples. Study participants from Meyerton were contacted for participation in the imaging portion of the study. Inclusion criteria included age ≥ 40 years and no contraindication to MRI [10]. Participants eligible for brain imaging in the present work were screened as a part of the original cohort study. Individuals were excluded from further participation if they had neurologic co-morbidities or were using a dopamine receptor blocking medication.

### 2.3. Clinical assessments

After completing the study questionnaire at home, participants were examined at a central location in the community by a single movement disorder specialist (B.A.R.) for signs of parkinsonism, using the UPDRS3, who was blinded to the cumulative Mn exposure of the participant. In addition to the UPDRS3 total score, we combined selected UPDRS3 subscores [5] as secondary clinical outcomes to evaluate for associations with specific clinical signs. If a full UPDRS3 could not be obtained secondary to injury, when possible, we imputed one or more missing subscores based on all participants with complete UPDRS3 subscores [10].

### 2.4. Toenail sample collection and analysis

A subset of the participants agreed to provide toenail samples (N = 19). We clipped small and big toenail samples separately and washed all samples once with 1 % Triton-X100 in an ultrasonic bath for 20 min and five times with ultrahigh quality water, dried overnight at 60°C, weighed, and digested in 1 mL of 65 % Suprapur^®^ nitric acid. We used environmental scanning electron microscope (ESEM, Quattro, Thermo Fisher Scientific) imaging to visually determine whether the washing procedure substantially removed exogeneous contamination.

We quantified metal content in toenail samples using an inductively coupled plasma-mass spectrometer (NexION^®^ 2000, PerkinElmer, Norwalk, CT). Recovery from toenails, determined using spiked matrix blanks due to lack of suitable reference materials, was 98.6 ± 4.2 % for Mn. The detection limit was 2.0 μg/g for toenail Mn. For each participant, we obtained toenail metal abundances by computing the mass-weighted average from separately measured small and big toenail samples.

### 2.5. Environmental Mn exposure assessment

The Meyerton smelter facility’s footprint is 4.3 km^2^ and includes multiple furnaces. Only the West furnace was operating during this study period. We modeled emissions from this furnace using the Industrial Source Complex Short Term (ISCST3) Gaussian plume dispersion model. Dispersion modeling of stack emissions was conducted for each hour from October 2015 to December 2018, using hourly meteorology data from the Vaal Triangle Airshed network station in Three Rivers. We used wind speed and solar radiation data to assign atmospheric stability classes and stack physical parameters reported for the furnace. Mn stack emissions data were not available, so we modeled a unit mass emission rate and, for each receptor (nodes on a square grid extending 5000 m from the furnace with receptors at 100 m intervals and at 2 m height), we scaled the estimated ambient concentration to the estimated maximum ambient concentration across the modeling domain. The nominally three-year mean scaled concentrations at each receptor were spatially interpolated to estimate the value at each participant’s geocoded residence(s). Finally, we multiplied this exposure intensity by duration at the respective residence and summed all products across the residential history for each participant, as a measure of cumulative Mn exposure. We also calculated the distance from their residence to the smelter.

### 2.6. Magnetic resonance imaging (MRI) acquisitions

We acquired a 3-D magnetization-prepared rapid gradient echo (MPRAGE) image on each participant using a GE 1.5 T Signa HD23 16 channel MRI located in Vereeniging, South Africa. Sequences included a T1-weighted magnetization-prepared rapid gradient echo (MPRAGE (repetition time [TR]: 8500 ms; echo time [TE]: 3.1 ms; inversion time [TI]: 450 ms; field of view [FOV]: 24 × 19.2 cm; section thickness: 1.2 mm; matrix: 224 × 192; number of excitations [Nex]: 1.00) and standard T2-weighted sequences. All images were non-linearly registered to FMRIB58-FA standard-space image as a target image. Globus pallidus and white matter reference regions were extracted using hand drawn volumes of interest (VOIs) in standard atlas space. The pallidal VOIs outlined the entire structure, whereas the reference regions comprised a spherical pure frontal white matter region for the MRI. The VOIs were then projected onto the atlas aligned images of all participants; hence, VOI volumes were identical for all participants. The intensity of the signal in the VOI on the T1-weighted MPRAGE image was quantified by calculating an intensity index in the globus pallidus, also known as the pallidal index (PI), as follows: Pallidal Index = [(Left pallidal VOI+Right pallidal VOI)/(Left white matter control region+-Right white matter control region)]× 100 [12,18].

### 2.7. Statistical analyses

We performed all statistical analyses using Stata version MP 17.0. We first assessed demographic differences in age, sex, race, language, education, cigarette smoking, and toenail Mn; comparing participants who regularly consuming ≥ 3 drinks per week versus occasionally (<3 drinks per week) based upon the Centers for Disease Control and Prevention Dietary Guidelines for Alcohol [27]. We specifically included cigarette smoking and alcohol use as both are associated with PD [28,29] and alcohol consumption may have both direct and indirect effects on in-vivo Mn concentrations [30–32]. For these comparisons we used Pearson’s X^2^ (or Fisher’s exact test in variables for which there were any categories with <5 expected participants) for categorical variables and the non-parametric Mann-Whitney *U* test for continuous variables, including all measures of Mn exposure based on residential histories and toenail Mn.

For our primary comparisons, i.e., MRI measures in relation to both Mn exposure, and toenail Mn samples, and UPDRS3, we used multivariable linear regression. We visually assessed the association between PI and UDPRS3 scores and subscores using locally weighted scatterplot smoothing (LOWESS). We examined the relationship between the PI and cumulative Mn exposure, concentration of Mn exposure, residential distance to a Mn smelter, and toenail Mn each retained as continuous variables modeled linearly. We also assessed the relationship between the PI and UPDRS3 scores, with UPDRS3 scores or subscores as the dependent variable and PI as the independent variable. We applied inverse probability weighting [33] to all analyses except those including toenail samples to adjust for confounding by age at the time of the exam, sex, and MRI software update (pre/post June 2019). Age as of the exam date was included in the weights for all models given the known strong, positive association between age and both cumulative exposure and UPDRS3 scores [5]. Sex was included in all weights as it was selected as a confounder *a priori*. Additionally, as the probability of receiving an MRI prior to versus post software update in June 2019 varied across individuals, we accounted for this systematic difference in our inverse probability weights by including the binary variable for MRI software update to adjust for this potential bias. The IPW could not be applied to analyses including toenail samples due to the limited sample sizes. In these instances, we assessed associations with the MRI PI using multivariable linear regression using age, sex, and pre/post software update. There was no statistical difference between alcohol use or Mn exposure among participants that were scanned prior to versus after the software update.

Finally, we examined the effects of adjustment for smoking and alcohol use on the PI and UPDRS3, while implementing the inverse probability weighting previously described to account for other confounders. Regarding alcohol use, we performed two separate models, one where alcohol use was defined as a binary variable, and another model where alcohol use was a continuous variable. For the binary variable, we defined alcohol use as regularly consuming alcohol (3≥ drinks per week) versus occasionally (<3 drinks per week). For the continuous variable for alcohol, we used the total current grams per week of alcohol consumed. Cigarette smoking was a binary variable defined as reported ever or never use of cigarettes. In addition, we evaluated both cigarette usage and regular alcohol consumption as potential effect modifiers by testing for a statistical interaction using a product term while retaining the main effects terms in the model. For all analyses, we considered a two-sided P value of 0.05 as statistically significant, evidenced by the exclusion of zero from the 95 % confidence interval (CI) for the β coefficient.

## 3. Results

### 3.1. Characteristics of participants

Cumulative Mn exposure, the UPDRS3 total scores, and a majority of the subscores were all higher in participants who sporadically consumed alcohol when compared to the participants who regularly consumed alcohol (Table 1). There were no significant differences in age, the concentration of Mn exposure, distance from residence to the smelter, time at residence, race, primary language, or education between the groups. Toenail Mn levels were similar between sporadic and regular alcohol consumption groups. There were more men and cigarette smokers in the participants who regularly consumed alcohol (Table 1).

### 3.2. MRI pallidal indices, toenail Mn, and residential exposure metrics

There was no clear association between the PI and Mn exposure metrics while applying inverse probability weights (data not shown). Higher toenail Mn was associated with a higher PI (β = 1.66, 0.19, 3.12, p = 0.03) after adjusting for age, sex, and pre/post software update. Toenail Mn was also inversely associated with distance from residence to smelter (ρ= −0.52, p = 0.03).

### 3.3. Clinical associations with the MRI pallidal index

**Fig. 1.**
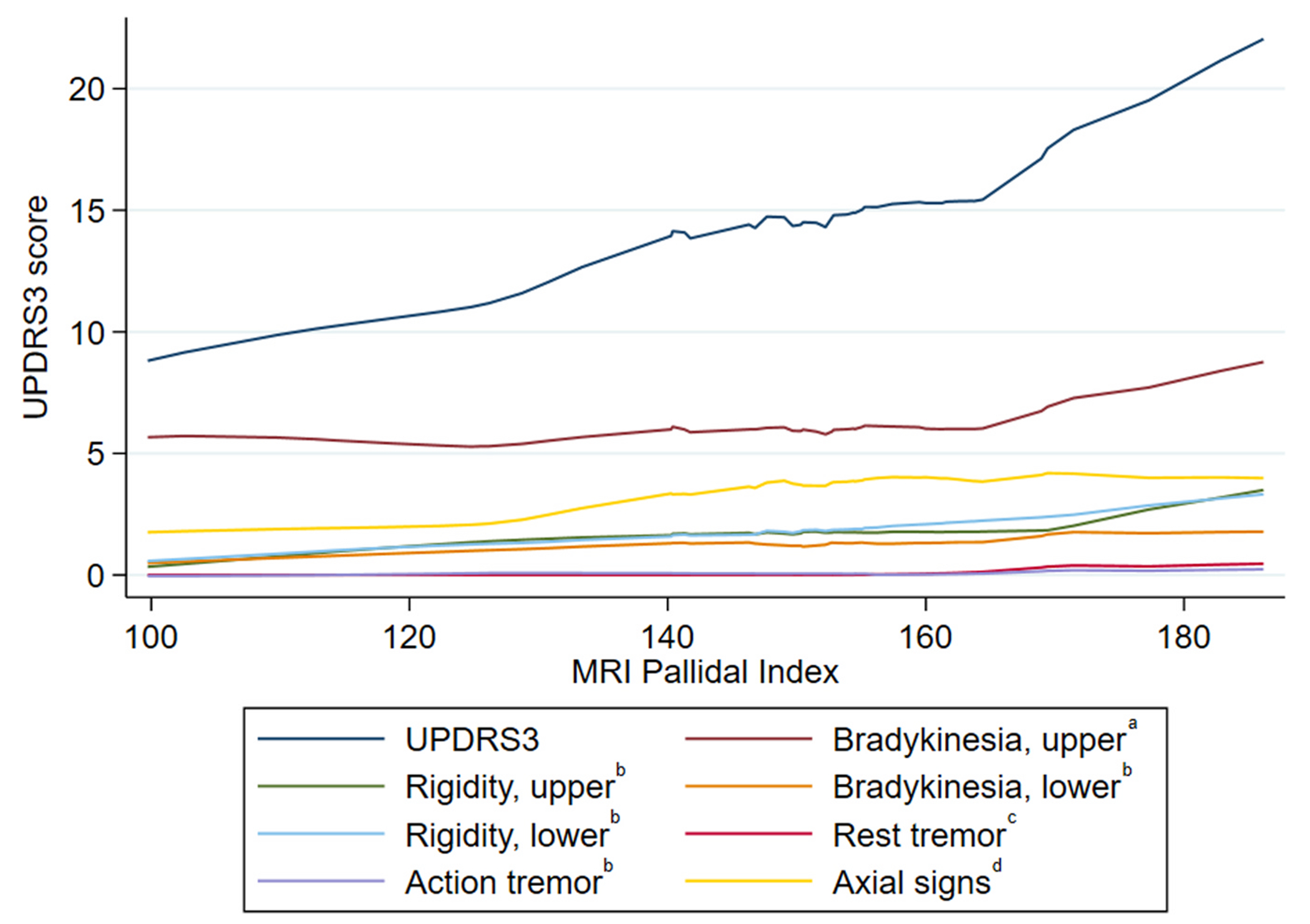
The relationship between MRI pallidal index and the Unified Parkinson’s Disease Rating Scale motor subsection 3 scores (N = 53), Gauteng, South Africa, 2016–2020. There is a positive relationship between the pallidal index and the total Unified Parkinson’s Disease Rating Scale motor subsection 3 (UPDRS3) score and axial signs^d^ as depicted by locally weighted scatterplot smoothing (LOWESS). ^a^ Sum of six UPDRS3 subscores: Finger-tapping, hand rotations, and rapid arm movements for each limb. ^b^ Sum of the two UPDRS3 subscores (one for each limb). ^c^ Sum of five UPDRS3 subscores: Upper limbs, lower limbs, and face. ^d^ Sum of eight UPDRS3 subscores: Speech, facial expression, neck rigidity, difficulty arising from a chair, posture, gait, postural instability, and global bradykinesia.

**Fig. 2.**
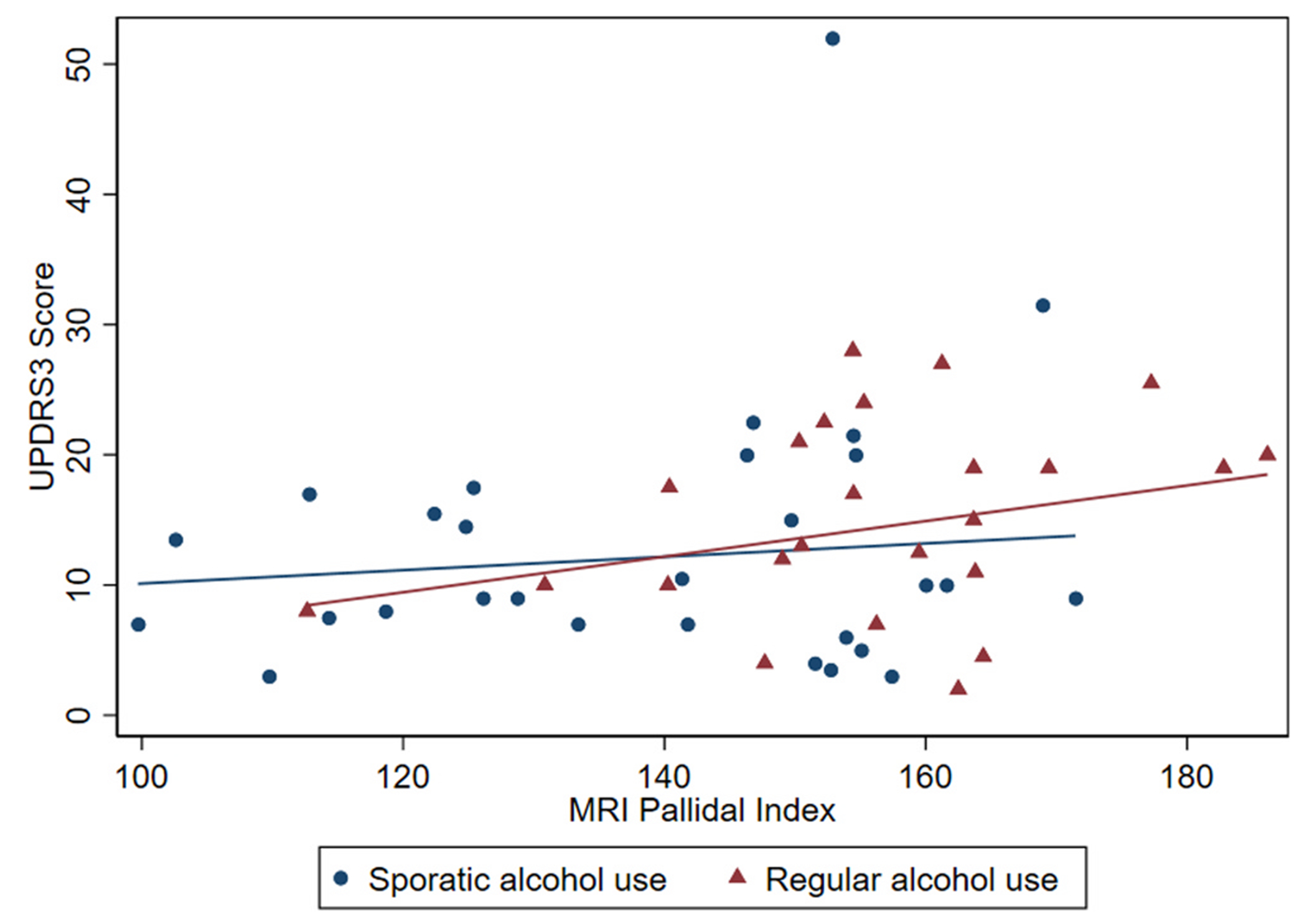
The relationship between MRI pallidal index and the Unified Parkinson’s Disease Rating Scale motor scores by alcohol consumption (N = 53), Gauteng, South Africa, 2016–2020. There is a positive relationship between the pallidal index and the total Unified Parkinson’s Disease Rating Scale motor subsection 3 (UPDRS3) score in the Mn exposed participants who regularly consumed alcohol that is not present in those with only occasional alcohol use.

PI was associated with the total UPDRS3 scores (β = 0.10, 0.01, 0.18) and the axial signs subscores (β = 0.03, CI 0.003, 0.06) (Table 2, Fig. 1). There were also strong trends in the associations between the PI and subscores for upper limb rigidity (β = 0.02, CI −0.005, 0.04) and lower limb rigidity (β = 0.02, CI −0.001, 0.04). When alcohol was assessed as an effect modifier on the relationship between PI and UPDRS3, a marked positive association was observed between the PI and total UPDRS3 among those who regularly consumed alcohol and no association was suggested between PI and total UPDRS3 among those who consumed alcohol only occasionally, although the interaction term was not significant (p_interaction_=0.34), (Table 3, Fig. 2). The UPDRS3 subscore for lower limb rigidity also demonstrated a positive association between PI and total UPDRS3 in those who regularly consumed alcohol and was not observed for those who occasionally consumed alcohol, with a significant interaction term (p_interaction_=0.01) (Table 3). There was not a material change in PI and UPDRS3 when adjusting for the total current grams of alcohol consumed per week (β = 0.08, CI −0.02, 0.18; p_interaction_=0.59). Adjustment for cigarette smoking (as a main effect) did not affect these relationships.

## 4. Discussion

This study demonstrates an association between the PI as a marker of in-vivo Mn deposition and parkinsonism in participants from a large residential cohort with an average ambient PM_2.5_-Mn exposure of up to 215 ng/m^3^ [10], much lower than previous studies of occupationally exposed cohorts [5,10,34,35]. In addition, these ambient levels are well below the United States Environmental Protection Agency (EPA) lowest observed adverse effect level (LOAEL) of 50,000 ng/m^3^ for PM_2.5_-Mn, indicating direct brain toxicity at exposures substantially lower than the current accepted LOAEL [36,37]. This has important potential public health implications as individuals who live around Mn-emitting facilities, not just those working within the plant, appear at risk for adverse neurologic outcomes. This also emphasizes MRI is a particularly useful tool to detect brain effects in lower level exposures as hyperintense T1-weighted signal can be detected among exposed individuals with no or minimal signs or symptoms of Mn neurotoxicity [38,39].

Mn neurotoxicity in humans occurs through two primary mechanisms of action: excessive exposure to airborne Mn [5,6] and failure of elimination secondary to severe liver dysfunction [32]. Interestingly, a recent study in mice demonstrated alcohol exposure induced a dose-dependent increase in brain Mn levels secondary to increased iron transporters which assist in Mn transportation into the brain [30]. This suggests alcohol consumption may have a direct effect on brain Mn deposition. In our study, we observed an effect of alcohol use on the relationship between the PI and both total UPDRS3 scores and the subscore for lower limb rigidity in this Mn exposed community. Lower limb rigidity was also a prominent sign in our previous study documenting dose-dependent progression of parkinsonism in occupational Mn exposure suggesting it may be a key clinical characteristic of Mn neurotoxicity [5]. Among participants who regularly consumed alcohol, we observed a significant, positive relationship between the PI and UPDRS3 scores compared to no significant relationship among participants who consumed alcohol only occasionally. Interestingly, Ellingsen et al. observed a similar interaction between Mn exposure and alcohol on neurobehavioral outcomes in occupationally exposed welders [40]. These observations suggest alcohol and Mn exposure exhibit synergistic neurotoxic effects in humans. The underlying mechanism may be secondary to alcohol enhancing brain Mn deposition; however, this hypothesis would require further investigation outside the scope of the current study.

The combined effects of Mn exposure and alcohol consumption are particularly relevant to groups or countries with large socioeconomically disadvantaged populations, as is the case in South Africa. Lower socioeconomic status (SES) communities experience higher exposures to air pollutants and an increased susceptibility to poor health resulting in adverse health effects secondary to environmental factors [25]. Globally, socioeconomically disadvantaged populations also exhibit different alcohol consumption patterns and are more likely to experience greater harm from alcohol consumption when compared to those from middle and higher socioeconomic populations [41,42]. Our study suggests that the combination of industrial air pollution containing Mn with regular alcohol consumption may lead to worse neurological outcomes in populations with both higher exposures to air pollutants and higher rates of alcohol consumption. However, this also suggests the potential for direct public health interventions regarding both emission regulations and alcohol consumption which could be implemented to improve neurologic health outcomes.

### 4.1. Limitations

This study has several potential limitations. First, not everyone invited to participate was willing to participate in the overall cohort or the imaging sub-study. Second, industrial emissions contain a number of elements and gases [43]; therefore, we cannot fully exclude a contribution from other neurotoxicants present in airborne emissions. However, the PI was correlated with toenail Mn within this cohort and bilateral increased T1 weighted pallidal signal is a characteristic finding of Mn neurotoxicity. Alternative causes are limited to Wilson’s disease (copper), hepatic encephalopathy, acquired non-Wilsonian hepatocerebral degeneration, global hypoxia, methemoglobin, calcifications, and hamartomas [44]. Wilson’s disease is extremely rare, and hypoxia, methemoglobin, calcifications, and hamartomas have their own additional identifying imaging characteristics. Chronic, high levels of alcohol consumption can lead to cirrhosis/liver failure and subsequent hepatic encephalopathy or acquired non-Wilsonian hepatocerebral degeneration (secondary to a failure to clear Mn) however none of the imaging participants had a history of cirrhosis or liver failure making these unlikely to be the sole cause of the elevated PIs. Lastly, the total number of participants within this imaging study is relatively small but the uniqueness of the population, underrepresentation of minority groups and cultures in the current literature, and the challenging nature of imaging studies within an environment with scarce resources offset this limitation.

## 5. Conclusion

In summary, we demonstrated an association between the T1-weighted PI and parkinsonism in an environmental Mn-exposed cohort, indicating in-vivo neurotoxicity. This association is amplified by the regular consumption of alcohol.

## Figures and Tables

**Table 1:** Characteristics of participants undergoing MRI by alcohol use, from Gauteng, South Africa, 2016–2020.

	Sporadic alcohol consumption^a^ N = 29	Regular alcohol consumption^a^ N = 24	

	n (%)	n (%)	p-value^b^
Sex			
Female	25 (86.2)	9 (37.5)	<0.0001
Male	4 (13.8)	15 (62.5)	
Race			
Black	29 (100)	24 (100)	0.53
Language			
Sesotho	17 (58.6)	15 (62.5)	0.88
IsiXhosa	7 (24.1)	6 (25.0)	
IsiZulu	4 (13.8)	3 (12.5)	
Setswana	0 (0.0)	0 (0.0)	
Sepedi	0 (0.0)	0 (0.0)	
Other	1 (3.5)	0 (0.0)	
Education^c^			
None/non-formal schooling	6 (20.7)	5 (20.8)	0.95
Primary	12 (41.4)	9 (37.5)	
Secondary	9 (31.0)	7 (29.2)	
Matric or higher	2 (6.9)	3 (12.5)	
Smoking cigarettes			<0.0001
Never	28 (96.6)	11 (45.8)	
Ever	1 (3.4)	13 (54.2)	
	Mean (SD)	Mean (SD)	p-value^b^
Age, years	56.5 (11.2)	54.5 (9.5)	0.53
Minimum	40.4	40.8	
Median	56.9	54.0	
Maximum	89.9	73.7	
Cumulative Mn exposure, years	2.10 (0.93)	1.63 (0.87)	0.05
Minimum	0.28	0.25	
Median	1.97	1.39	
Maximum	4.04	3.54	
Time at current address, years	52.8 (10.3)	47.6 (14.9)	0.21
Minimum	21.1	7.6	
Median	53.7	48.0	
Maximum	66.7	65.8	
Mn Concentration, 3-year mean^d^	0.14 (0.07)	0.12 (0.07)	0.36
Minimum	0.04	0.04	
Median	0.14	0.11	
Maximum	0.30	0.26	
Distance to Mn smelter, km	1.60 (0.68)	1.77 (0.76)	0.46
Minimum	0.85	0.95	
Median	1.37	1.58	
Maximum	3.15	3.01	
Toenail Mn, μg/g^e^	11.3 (4.6)	12.1 (7.0)	0.78
Minimum	5.5	1.7	
Median	11.4	11.9	
Maximum	20.9	22.9	
UPDRS3 total	13.1 (10.2)	15.4 (7.4)	0.10
Minimum	3	2	
Median	10	16	
Maximum	52	28	

**Abbreviations:** Mn=manganese; SD=standard deviation.

a Regular consumption defined as ≥ 3 drinks per week or occasional as < 3 drinks per week.

b P-value determined using Fisher’s exact test due to categories with small, expected numbers. The p-value for age, cumulative Mn exposure, Mn concentration, distance to Mn smelter, and total UPDRS3 were determined using a Mann-Whitney *U* test.

c Primary is grades 1–7, secondary is grades 8–11, and matric is grade 12.

d The values are unitless ratios; ambient Mn concentrations (nominally 3-yr average) estimated by a dispersion model were normalized by the estimated maximum value across the modeled domain.

e Toe Mn analysis limited to 19 participants due to missing data. Analysis excludes 34 participants (sporadic alcohol consumers n = 18 (52.9 %); regular alcohol consumers n = 16 (47.1 %)

**Table 2:** Association between MRI pallidal index and the Unified Parkinson’s Disease Rating Scale motor subsection 3 scores (N = 53), Gauteng, South Africa, 2016–2020.

	Difference in UPDRS3 for each unit of MRI pallidal index (95 % CI)^a^
UPDRS3, total score	0.10 (0.01–0.18)*
UPDRS3 subscores	
Upper limb bradykinesia^b^	0.02 (−0.02, 0.06)
Upper limb rigidity^c^	0.02 (−0.005, 0.04)
Lower limb bradykinesia^c^	0.01 (−0.01, 0.02)
Lower limb rigidity^c^	0.02 (−0.001, 0.04)
Rest tremor^d^	0.003 (−0.003, 0.01)
Action tremor^c^	0.001 (−0.003, 0.003)
Axial signs^e^	0.03 (0.003–0.06)*

**Abbreviations:** CI=confidence interval; MRI=magnetic resonance imaging; UPDRS3 =Unified Parkinson’s Disease Rating Scale motor subsection part 3.

* Indicates a p-value ≤ 0.05

a β-coefficient estimates from linear regression with UPDRS3 total and subscores, each as outcome variables, and MRI pallidal index as a continuous independent variable. Each model is weighted to account for age, sex, and the MRI scan date (pre/post June 2019).

b Sum of six UPDRS3 subscores: Finger-tapping, hand rotations, and rapid arm movements for each limb.

c Sum of the two UPDRS3 subscores (one for each limb).

d Sum of five UPDRS3 subscores: Upper limbs, lower limbs, and face.

e Sum of eight UPDRS3 subscores: Speech, facial expression, neck rigidity, difficulty arising from a chair, posture, gait, postural instability, and global bradykinesia.

**Table 3:** Association between MRI pallidal index and the Unified Parkinson’s Disease Rating Scale motor subsection 3 scores, by alcohol use (N = 53), Gauteng, South Africa, 2016–2020.

	Difference in UPDRS3 for each unit of MRI pallidal index (95 % CI)^a^	

	Regular alcohol use^a^ (N = 24)	Occasional alcohol use (N = 29)
UPDRS3, total score	0.14 (0.03, 0.25)*	0.05 (−0.09, 0.20)
UPDRS3 subscores		
Upper limb bradykinesia^c^	0.04 (−0.01, 0.09)	0.01 (−0.05, 0.07)
Upper limb rigidity^d^	0.03 (−0.0001, 0.07)	−0.01 (−0.03, 0.02)
Lower limb bradykinesia^d^	0.005 (−0.01, 0.02)	0.01 (−0.02, 0.03)
Lower limb rigidity^d^	0.04 (0.005, 0.07)*	−0.001 (−0.03, 0.02)
Rest tremor^d^	0.01 (−0.01, 0.02)	–
Action tremor^e^	0.0001 (−0.01, 0.01)	–
Axial signs^f^	0.02 (−0.02, 0.05)	0.04 (−0.01, 0.09)

**Abbreviations:** CI=confidence interval; MRI=magnetic resonance imaging; UPDRS3 =Unified Parkinson Disease Rating Scale motor subsection part 3.

* Indicates a p-value ≤ 0.05

a β-coefficient estimates from linear regression with UPDRS3 total and subscores, each as outcome variables, and MRI pallidal index as a continuous independent variable. Each model is weighted to account for age, sex, and the MRI scan date (pre/post June 2019).

b Regular consumption defined as ≥ 3 drinks per week or occasional as < 3 drinks per week.

c Sum of six UPDRS3 subscores: Finger-tapping, hand rotations, and rapid arm movements for each limb.

d Sum of the two UPDRS3 subscores (one for each limb).

e Sum of five UPDRS3 subscores: Upper limbs, lower limbs, and face.

f Sum of eight UPDRS3 subscores: Speech, facial expression, neck rigidity, difficulty arising from a chair, posture, gait, postural instability, and global bradykinesia.

## Data Availability

Data from research participants in this study, who authorized sharing of their research data, will be made available to investigators with appropriate expertise and research support, after publication of the primary aims of this study. All shared data will be de-identified and will be released in accordance with U.S. and South African regulations.
